# Relationship Between BMI and Prediabetes in Chinese Adults: A Cross-Sectional Analytical Study

**DOI:** 10.1155/ije/5524318

**Published:** 2025-10-13

**Authors:** Beibei Luo, Wenbo Xu, Dan Ye

**Affiliations:** Clinical Laboratory, People's Hospital of Yuxi City, The Sixth Affiliated Hospital of Kunming Medical University, 21 Nieer Road, Yuxi, Yunnan 653100, China

**Keywords:** body mass index, prediabetes, prospective study

## Abstract

**Introduction:**

Prediabetes represents a critical stage in the progression towards diabetes. However, there is a scarcity of studies examining the specific impact of body mass index (BMI) on prediabetes risk among the Chinese population. This study aims to analyze the association between BMI and the risk of prediabetes in Chinese adults.

**Methods:**

In this cross-sectional analytical study, we analyzed data from 11,847 participants in the China Health and Retirement Longitudinal Study (CHARLS) conducted in 2011. For both univariate and multivariate analyses, logistic regression models were employed. Using a BMI range of 18.5–23.9 kg/m^2^ as the reference, we calculated the odds ratio (OR) and 95% confidence interval (95% CI) for different BMI categories and their associated outcomes.

**Results:**

Significant differences were observed in the distribution of variables such as gender, age, education level, marital status, smoking and drinking habits, systolic blood pressure (SBP), diastolic blood pressure (DBP), waist circumference (WC), total cholesterol (TC), triglycerides (TG), high-density lipoprotein cholesterol (HDL-C), low-density lipoprotein cholesterol (LDL-C), fasting plasma glucose (FPG), glycated hemoglobin (HbA1c), hypertension, dyslipidemia, heart disease, kidney disease, and prediabetes across different BMI groups (*p* < 0.05). Furthermore, when BMI was treated as a continuous variable, curve fitting analysis indicated that the risk of prediabetes increased when BMI exceeded 22.9 kg/m^2^.

**Conclusion:**

Obesity is a significant risk factor for prediabetes, with the prevalence of prediabetes increasing among overweight and obese individuals in China.

## 1. Introduction

Prediabetes represents a critical stage in the progression towards diabetes, characterized by blood sugar levels that are higher than normal but not yet in the diabetic range. It is one of the most prominent yet often overlooked public health concerns [1]. According to the Global Diabetes Map (10th Edition) published by the International Diabetes Federation 2021, the prevalence of diabetes among adults in China is 12.8%, affecting over 130 million individuals, with approximately 350 million people in a prediabetic state [2]. Furthermore, epidemiological studies indicate that more than 50% of adults in China are in a prediabetic condition [3].

The prevalence of prediabetes in China is rapidly increasing, largely due to changes in lifestyle, an aging population, and rising obesity rates [4]. The proportion of elderly individuals in China is growing, and with advancing age, pancreatic islet function declines, resulting in a reduced capacity for blood sugar regulation, thereby increasing the risk of prediabetes [5]. Additionally, older adults are often affected by multiple chronic conditions, such as hypertension and hyperlipidemia, which further elevate the risk of prediabetes [6]. The obesity rate in China is also escalating annually [7]. Obesity can induce insulin resistance [8], a precursor to prediabetes, and is closely linked to various metabolic disorders [9], including hypertension and hyperlipidemia, which compound the risk of developing prediabetes.

There is a limited number of studies examining the specific impact of body mass index (BMI) on the risk of prediabetes in China. Understanding the varying relationship between obesity and prediabetes risk is crucial for public health, particularly for developing effective screening protocols and intervention strategies. Utilizing data from the 2011 China Health and Retirement Longitudinal Study (CHARLS), this study analyzed the association between BMI and prediabetes risk among Chinese adults. It also provided targeted recommendations for the prevention and management of prediabetes and diabetes, offering data-driven support for government policies aimed at disease control.

## 2. Methods

### 2.1. Cross-Sectional Analysis and Participants

**Figure 1:**
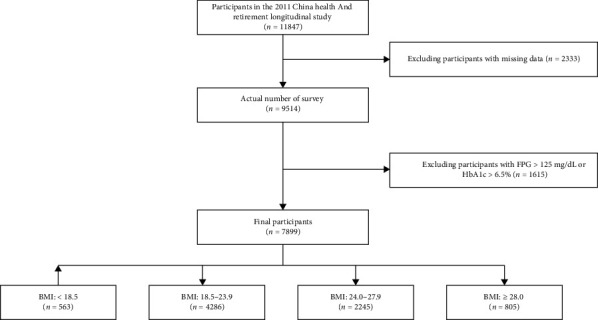
Flowchart of the study participants.

CHARLS is a series of nationally representative, periodic cross-sectional surveys targeting families and individuals aged 45 and above in China, initiated in 2011. The survey encompasses a wide range of data, including demographic details, family structure, financial support, health status, physical measurements, healthcare utilization, medical insurance, employment, retirement, pensions, income, consumption, assets, and community characteristics. For this study, data from 11,847 participants in the 2011 CHARLS survey were initially selected. After excluding 3948 participants due to incomplete or inconsistent baseline information, a total of 7899 participants were included in the final analysis (Figure 1). Ethical approval for all CHARLS waves was obtained from the Institutional Review Board at Peking University, with approval numbers IRB00001052-11015 for the main household survey, including anthropometrics and IRB00001052-11014 for biomarker collection. All respondents provided informed consent prior to participation. This study also received ethical clearance from the Ethics Committee of the Sixth Affiliated Hospital of Kunming Medical University (Approval No. 2024kmykdx6f134) and was conducted in accordance with the principles of the Declaration of Helsinki.

### 2.2. Anthropometric and Biochemical Measurements

All data used in this study were derived from the 2011 CHARLS, which included demographic information, records of complications, medication usage, biomarkers, and blood samples. BMI was calculated using height measured with a Seca 213 stadiometer and weight with an Omron HN-286 weighing scale. Waist circumference was assessed using a soft measuring tape placed 1 cm above the umbilicus. Blood pressure was measured three times consecutively with an Omron HEM-7112 sphygmomanometer after participants had rested quietly; the average value was recorded at intervals of 45 s [10]. Biochemical parameters, including total cholesterol (TC), triglycerides (TG), high-density lipoprotein cholesterol (HDL-C), low-density lipoprotein cholesterol (LDL-C), fasting plasma glucose (FPG), and glycosylated hemoglobin (HbA1c), were measured using standard methods at KingMed Laboratory. Specifically, TC and TG were assessed via the oxidase method, HDL-C and LDL-C by the direct method, FPG using the hexokinase method, and HbA1c through standard protocols [11].

### 2.3. Definitions of Prediabetes and Grouping

The American Diabetes Association defines prediabetes as having an HbA1c level between 5.7% and 6.4% or a FPG level between 100 mg/dL and 125 mg/dL [12]. According to Chinese guidelines, BMI is classified into four categories: underweight (BMI < 18.5), normal weight (BMI 18.5 to < 24.0), overweight (BMI 24.0 to < 28.0), and obesity (BMI ≥ 28.0) [13].

### 2.4. Statistical Analysis

Continuous variables with a normal or approximately normal distribution were described using the mean and standard deviation, and the F-test was used for group comparisons. For categorical data, the number of cases and the percentage distribution (*n*%) were reported, and the chi-square test was used for group comparisons. A logistic regression model was employed for both univariate and multivariate analyses. The odds ratio (OR) and 95% confidence interval (95% CI) were calculated using BMI categories, with a reference range of 18.5–23.9 kg/m^2^. The model selection process involved identifying the factors influencing prediabetes through univariate analysis. Based on a literature review and expert knowledge, relevant variables related to BMI and prediabetes were incorporated into the model. A fitting curve between BMI and prediabetes was then drawn to determine the critical threshold. Statistical significance was set at *p* < 0.05. All analyses were conducted using the R statistical software package (https://www.R-project.org, The R Foundation).

## 3. Results

### 3.1. Baseline Characteristics

A total of 7899 participants from CHARLS were included in the study. Significant differences were observed in the distribution of gender, age, education, marital status, smoking, alcohol consumption, systolic blood pressure (SBP), diastolic blood pressure (DBP), waist circumference (WC), TC, TG, HDL-C, LDL-C, FPG, HbA1c, hypertension, dyslipidemia, heart disease, kidney disease, and prediabetes across different BMI groups (*p* < 0.05) (Table 1).

### 3.2. Main Outcomes

**Figure 2:**
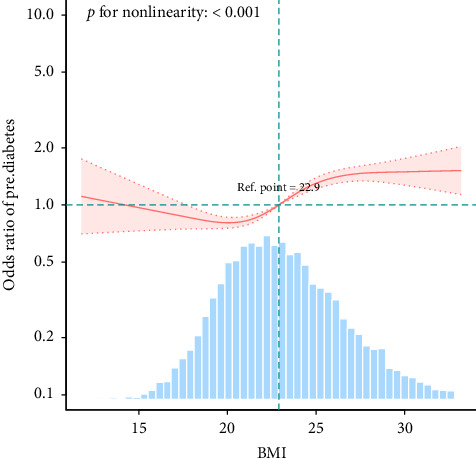
Fitting curve evaluating HbA1c for prediabetes.

After adjusting for age, sex, education, smoking, alcohol consumption, hypertension, dyslipidemia, heart disease, and kidney disease, overweight was associated with a 47% increased risk of prediabetes (OR 1.47, 95% CI 1.32–1.63), while obesity was associated with a 57% increased risk (OR 1.57, 95% CI 1.34–1.85) (Table 2). When BMI was treated as a continuous variable, curve fitting revealed that a BMI exceeding 22.9 kg/m^2^ was associated with an increased risk of prediabetes (Figure 2).

## 4. Discussion

This study aims to investigate the relationship between BMI and the risk of prediabetes, as well as the impact of obesity on the incidence of prediabetes among middle-aged and elderly individuals in China. The findings indicate that the risk of prediabetes is significantly higher in obese individuals compared to those with normal weight, and that overweight individuals also face an increased risk. Specifically, when BMI exceeds 22.9 kg/m^2^, the risk of prediabetes rises. As BMI increases, the risk of prediabetes also increases. Therefore, weight management is an effective strategy for preventing prediabetes.

In our study, the risk of prediabetes increases when BMI exceeds 22.9 kg/m^2^. However, this threshold does not align with the commonly accepted standard for overweight. This suggests that a lower BMI may be sufficient to trigger the risk of prediabetes in this population. Age may be a key contributing factor; as individuals age, metabolic function declines, and insulin sensitivity may decrease [14], thereby elevating the risk of diabetes. Consequently, middle-aged and elderly individuals, even with a normal BMI, may face a higher risk of prediabetes due to age-related factors. An animal study on mice also demonstrated that aging leads to changes in the metabolic system, impairing glucose processing and insulin response [15].

Obesity is not only a primary characteristic of insulin resistance [16] but also has a profound impact on metabolic health. Excessive fat accumulation, particularly in the abdominal area, disrupts normal insulin function, preventing effective glucose uptake by cells and leading to insulin resistance [17]. Obesity has become a critical determinant in the onset and progression of insulin resistance and Type 2 diabetes [18]. Changes in lifestyle, including increased consumption of high-calorie diets and decreased physical activity, have contributed to the rising prevalence of obesity, which in turn has significantly increased the incidence of insulin resistance and diabetes among adults [19].

In this study, obesity is identified as a risk factor for prediabetes, aligning with findings from numerous other studies. A study [4] involving 1660 Italian teenagers aged 2 to 19 demonstrated that an increase in BMI has a causal relationship with insulin levels, with the effects of obesity on insulin resistance detectable even in childhood. Additional evidence [20] further supports the causal impact of obesity on fasting insulin levels, particularly in relation to age- and gender-specific effects on cardiovascular risk factors. Moreover, obesity is a significant risk factor for many age-related diseases [21]. Therefore, weight loss has emerged as a key intervention for preventing prediabetes, particularly in reducing the associated risks [22].

This study focuses on prediabetes, a stage that has received limited attention in previous research. During this stage, although fasting blood glucose or postprandial blood glucose levels are elevated compared to healthy individuals, they do not yet meet the diagnostic criteria for diabetes [23]. If left unaddressed, prediabetes can gradually progress, ultimately leading to the development of Type 2 diabetes. However, prediabetes is not an irreversible condition. Active lifestyle interventions, particularly targeted dietary management and regular physical exercise, can significantly influence the disease's progression [24, 25]. Dietary interventions typically involve reducing the intake of high-sugar and high-fat foods while increasing the consumption of vegetables, whole grains, lean meats, and low-sugar fruits to optimize diet structure and reduce blood sugar load. Concurrently, regular aerobic exercise, such as brisk walking, swimming, and cycling, helps control weight, enhances insulin sensitivity, promotes the effective use of blood sugar, and slows the rise in blood glucose. Effective weight control is crucial in reversing the course of prediabetes. Therefore, for individuals with prediabetes, a combination of appropriate dietary control and moderate physical activity is an essential strategy to slow or even reverse the disease, thus reducing the risk of diabetes [26].

However, this study has several limitations. First, although the sample size is large, the majority of participants are middle-aged and elderly individuals, and the study does not include younger populations. Second, while most confounding factors were considered, the baseline survey did not capture all potential exposures, and some unmeasured variables (e.g., hormone levels, anxiety, and depression) were not included in the model adjustment, which may introduce residual confounding. Third, the absence of follow-up data limits the ability to assess the incidence trends of prediabetes and the impact of interventions such as diet and exercise on the progression to diabetes. These limitations suggest directions for future research on the incidence and determinants of prediabetes.

## 5. Conclusion

In conclusion, our findings demonstrate that obesity is a significant risk factor for prediabetes, with the risk increasing among overweight and obese individuals in China. Strengthening the prevention and control of obesity and overweight can effectively reduce the incidence of prediabetes and diabetes, thereby improving public health and quality of life.

## Figures and Tables

**Table 1:** Baseline characteristics of participants.

Variables	Total (*n* = 7899)	BMI (kg/m^2^)				*p*
		< 18.5 (*n* = 563)	18.5–23.9 (*n* = 4286)	24.0–27.9 (*n* = 2245)	≥ 28.0 (*n* = 805)	
Male, *n* (%)	3634 (46.0)	274 (48.7)	2230 (52)	889 (39.6)	241 (29.9)	< 0.001
Age	58.9 ± 9.7	64.4 ± 10.3	59.4 ± 9.8	57.4 ± 8.9	56.7 ± 8.8	< 0.001
Education, *n* (%)						< 0.001
Elementary school and below	3772 (47.8)	347 (61.6)	2102 (49)	977 (43.5)	346 (43)	
Elementary school	1784 (22.6)	112 (19.9)	995 (23.2)	489 (21.8)	188 (23.4)	
Middle school	1584 (20.1)	75 (13.3)	814 (19)	518 (23.1)	177 (22)	
High school and above	759 (9.6)	29 (5.2)	375 (8.7)	261 (11.6)	94 (11.7)	
Married, *n* (%)	6612 (83.7)	438 (77.8)	3535 (82.5)	1942 (86.5)	697 (86.6)	
Smoke, *n* (%)	3077 (39.0)	266 (47.2)	1915 (44.7)	700 (31.2)	196 (24.3)	
Drink, *n* (%)						< 0.001
Never	1966 (24.9)	138 (24.5)	1207 (28.2)	488 (21.7)	133 (16.5)	
Less than once a month	628 (8.0)	42 (7.5)	349 (8.1)	179 (8)	58 (7.2)	
More than once a month	5305 (67.2)	383 (68)	2730 (63.7)	1578 (70.3)	614 (76.3)	
SBP (mmHg)	129.2 ± 24.0	125.2 ± 22.8	126.9 ± 21.6	132.2 ± 28.3	135.7 ± 21.5	< 0.001
DBP (mmHg)	75.3 ± 12.7	71.2 ± 12.0	73.5 ± 12.3	77.7 ± 12.6	80.4 ± 12.7	< 0.001
Waist (cm)	83.6 ± 12.3	71.3 ± 8.2	79.4 ± 9.7	89.6 ± 9.5	97.8 ± 13.5	< 0.001
TC (mg/dL)	192.0 ± 37.5	186.6 ± 37.5	189.6 ± 36.8	195.9 ± 37.7	197.7 ± 39.2	< 0.001
TG (mg/dL)	121.0 ± 75.0	95.4 ± 52.0	109.9 ± 66.3	135.5 ± 79.0	157.9 ± 97.4	< 0.001
HDL-C (mg/dL)	52.1 ± 15.0	59.9 ± 16.1	54.4 ± 15.2	48.3 ± 13.1	44.6 ± 11.8	< 0.001
LDL-C (mg/dL)	116.8 ± 34.0	109.4 ± 32.4	114.4 ± 33.0	121.2 ± 34.6	122.0 ± 37.1	< 0.001
FPG (mg/dL)	100.0 ± 12.0	98.2 ± 12.4	99.1 ± 11.9	101.4 ± 11.7	102.3 ± 12.0	< 0.001
HbA1c (%)	5.1 ± 0.4	5.1 ± 0.4	5.1 ± 0.4	5.1 ± 0.4	5.2 ± 0.4	< 0.001
Hypertension, *n* (%)	1746 (22.2)	75 (13.4)	690 (16.2)	631 (28.3)	350 (43.6)	< 0.001
Dyslipidemia, *n* (%)	607 (7.9)	15 (2.7)	206 (4.9)	237 (10.8)	149 (19.1)	< 0.001
Heart problems, *n* (%)	849 (10.8)	60 (10.7)	383 (9)	275 (12.3)	131 (16.4)	< 0.001
Kidney disease, *n* (%)	513 (6.5)	50 (9)	287 (6.7)	132 (5.9)	44 (5.5)	0.037
Prediabetes, *n* (%)	3903 (49.4)	320 (56.8)	2276 (53.1)	974 (43.4)	333 (41.4)	< 0.001

*Note:* TG, triglycerides; HDL-C, high-density lipoprotein; LDL-C, low-density lipoprotein; HbA1c, glycated hemoglobin.

Abbreviations: BMI, body mass index; DBP, diastolic blood pressure; FPG, fasting plasma glucose; SBP, systolic blood pressure; TC, Total cholesterol.

**Table 2:** Relationship between BMI and prediabetes.

	BMI (kg/m^2^)				*p*
	18.5–23.9 (*n* = 4286)	< 18.5 (*n* = 563)	24.0–27.9 (*n* = 2245)	≥ 28.0 (*n* = 805)	
Unadjusted model	1.00 (Ref)	0.86 (0.72–1.03)	1.48 (1.33–1.64)	1.6 (1.38–1.87)	< 0.001
Model I OR (95% CI)	1.00 (Ref)	0.79 (0.66–0.95)	1.54 (1.38–1.71)	1.7 (1.45–1.98)	< 0.001
Model II OR (95% CI)	1.00 (Ref)	0.8 (0.66–0.95)	1.53 (1.38–1.7)	1.69 (1.45–1.98)	< 0.001
Model III OR (95% CI)	1.00 (Ref)	0.8 (0.66–0.96)	1.47 (1.32–1.63)	1.57 (1.34–1.85)	< 0.001

*Note:* Model I: Adjusted for gender + age; Model II: adjusted for Model I + education + smoke + drink; Model III: adjusted for Model II + hypertension + dyslipidemia + heart problems + kidney disease.

Abbreviations: BMI, body mass index; OR, odds ratio.

## Data Availability

The data analyzed in this study are publicly available from the CHARLS dataset (https://charls.pku.edu.cn/). All data were accessed and downloaded in accordance with CHARLS's data use agreement.
